# *Toxoplasma gondii* ROP16_I_ Deletion: The Exacerbated Impact on Adverse Pregnant Outcomes in Mice

**DOI:** 10.3389/fmicb.2019.03151

**Published:** 2020-01-31

**Authors:** Wen Cui, Cong Wang, Qingli Luo, Tian Xing, Jilong Shen, Wei Wang

**Affiliations:** ^1^Department of Pathogen Biology, Provincial Laboratories of Pathogen Biology and Zoonoses Anhui, School of Basic Medicine, Anhui Medical University, Hefei, China; ^2^Department of Clinical Laboratory, The Second Hospital of Hefei, Hefei, China; ^3^The Key Laboratory of Oral Disease Research of Anhui, College and Hospital of Stomatology, Anhui Medical University, Hefei, China; ^4^Department of Clinical Laboratory, The First Affiliated Hospital of Anhui Medical University, Hefei, China

**Keywords:** *Toxoplasma gondii*, ROP16, macrophage, immune tolerance, adverse pregnant outcome

## Abstract

Imbalance of Th1 and Th2 response at the maternal–fetal interface is considered as a radical event in the pathogenesis of immunity-related pregnant diseases. It has been demonstrated that the ROP16_I_, a rhoptry protein of *Toxoplasma gondii*, and the viable parasite with ROP16_I_ may induce M2 macrophage polarization in host innate immunity and may be involved in the adverse pregnant outcomes. However, the mechanisms by which *T. gondii*-derived effectors subvert the immune tolerance in the pathology of pregnancy remain unclear. Here, we constructed the RH strain with ROP16_I_ deletion (RHΔ*rop16*) to explore the pathogenesis of abnormal pregnancy. We found that C57BL/6 mice infected with RHΔ*rop16* exhibited the increased resorption of fetuses and more severe adverse pathology of placentae at the early phase of gestation, as compared to the mice infected with RH wild type (RH WT) parasite. Additionally, RHΔ*rop16* strain infection significantly promoted M1 macrophage phenotypes of CD80 and CD86, and decreased CD206 expression of M2 macrophages, with upregulation of the iNOS and downregulation of the Arg-1 expression in placental homogenates. Simultaneously, the pro-inflammatory cytokines of IL-12 and TNF-α were elevated whereas the anti-inflammatory cytokine of TGF-β1 was dampened. Moreover, the p38α mitogen-activated protein kinase (p38α MAPK) was notably phosphorylated in placental macrophages infected with both RHΔ*rop16* and RH WT strains compared with the control. Taken together, our findings indicated that ROP16_I_ deletion of type I RH strain may cause exacerbated adverse pregnant outcomes, which is attributable to subversion of the maternal immune tolerance due to the increased pro-inflammatory cytokines in the pregnant animals. The results also suggest that ROP16_I_ might be a protective factor and other *T. gondii*-derived molecules might be involved in the M1–Th1 biased pathological process in aberrant pregnancy at the early phase of gestation.

## Introduction

*Toxoplasma gondii* is an opportunistic food-borne protozoon with an extraordinarily broad host range of all warm-blooded animals, including humans (Montoya and Liesenfeld, 2004; Leng et al., 2009). Humans and animals are infected by ingesting food that contains cysts or water that is contaminated with oocysts of *T. gondii* (Black and Boothroyd, 2000; Yarovinsky, 2014). It is estimated that over one billion people are chronically infected with this parasite worldwide, although the data of regional investigations vary considerably (Montoya and Liesenfeld, 2004; Dubey, 2009). *T. gondii* infection is usually asymptomatic in immunocompetent individuals but may result in severe consequences in immunocompromised people (e.g., patients with AIDS, organ transplantation, or cancers) (Nissapatorn, 2009; Luma et al., 2013; Schmidt et al., 2013). Importantly, vertical transmission of *T. gondii via* placenta may cause abortion, constituting a serious threat to humans and leading to great loss of livestock production (Montoya and Remington, 2008; Hide et al., 2009). Initial infection of women with *T. gondii* during pregnancy, particularly in the first trimester, may cause miscarriage and preterm birth and increase the susceptibility of fetuses to toxoplasmosis resulting in hydrocephaly, microcephaly, intracranial calcification, and even loss of life (Pfaff et al., 2007; Robbins et al., 2012). The variability of disease severity in infected host is linked to the genetic structure of *T. gondii* strains and to the exposure burden of the parasite (Saeij et al., 2006; Melo et al., 2011; Hunter and Sibley, 2012; Shwab et al., 2014).

*Toxoplasma gondii* isolates from Europe and North America mostly belong to types I (RH and GT1), II (PRU and ME49), and III (CTG) (Lehmann et al., 2006; Shwab et al., 2014), but those from China present a dramatic difference in genetic structure and virulence, termed as type Chinese 1 (Wang et al., 2013). Studies have revealed that the majority of *T. gondii* isolates causing congenital toxoplasmosis in Europeans possess the feature of type II phenotype whereas type I strains are the most prevalent in Spanish people (Fuentes et al., 2001).

Macrophages can be roughly categorized into two types: classically activated macrophage (M1) and alternatively activated macrophage (M2). As antigen-presenting cells, macrophages have cross-talk between innate immunity and adaptive immunity. *T. gondii* parasite was found to preferentially invade macrophage/DC lineage cells during *in vivo* infection (Courret et al., 2006). Decidual immune cell populations approximately consist of 20% of macrophages that play a critical part in maintaining normal pregnancy. It has been well recognized that M2 macrophages are responsible for sustaining the normal microenvironment of pregnancy at the maternal–fetal interface (Nagamatsu and Schust, 2010). Actually, any subversion of M1/M2 macrophage balance may lead to pregnant disorders, such as pregnant loss, premature birth, or fetal growth restriction (Renaud and Graham, 2008; Brown et al., 2014).

*Toxoplasma gondii* establishes the long-lasting infection in host and has the developed sophisticated ways to manipulate host immunity. For instance, the parasite delivers effector proteins, which are released from the contents of rhoptries, micronemes, and dense granules of *T. gondii*, into host cells (Jensen et al., 2011; Rosowski et al., 2011; Hunter and Sibley, 2012). The secreted rhoptry proteins (ROPs) and dense granule proteins (GRAs) are characterized to hijack cell-signaling pathways of the host cells, which manipulate host immune response, and therefore determine parasite virulence and infection consequences (Jensen et al., 2011; Hakimi et al., 2017). Interestingly, ROP16 (the member of ROPs) gets injected into the host cell nucleus where it activates signal transducer and activator of transcription Stat3 and Stat6, initiating the signaling cascade of host cells. ROP16_I_ of type I strains, rather than ROP16_II_ of type II strains, decides ROP16 kinase activity on Stat3 and Stat6 (Yamamoto et al., 2009). Phosphorylation of Stat3 and Stat6 triggers anti-inflammatory response and M2 macrophage activation, which plays a crucial role in modulating host immunity at the early phase and is associated with the consequences of infection at the late phase. Contrarily, the GRA15_II_ directly activates the NF-κB of the host cells, promotes its rapid nuclear transcription, and drives the macrophage to M1 polarization (Rosowski et al., 2011). ROP16_I_ and GRA15_II_ can work independently of pattern recognition receptors to achieve M1 or M2 polarization (Jensen et al., 2011). Intriguingly, the WH3 strain of type Chinese 1, a predominant genotype in China, carries both key effectors: ROP16_I_ and GRA15_II_. Our previous study showed that the WH3Δ*rop16* strain with GRA15_II_ background of type Chinese 1 evoked the Th1- and Th17-biased response, leading to subversion of immune tolerance at the maternal–fetus interface and adverse pregnant outcomes (Wang et al., 2018). Recent studies have shown that RHΔ*rop16* infection in mice may cause serious ocular toxoplasmosis in the immune-privileged microenvironment, which is far away from the proliferation sites of the parasite, suggesting that the RH strain, with ROP16_I_ deficiency, remains a high pathogenesis of severe retinopathy in animal model (Rochet et al., 2019).

It has been known that vertical transmission of viable *T. gondii* parasite is a crucial route in aberrant pregnancy. However, we tried to identify the parasite by *Toxo*-DNA detection and bioassay in the maternal placental tissues with positive antibodies against *T. gondii* and abnormal pregnancies, but failed (data not published). Previous studies revealed that pregnant mice of IL-10 deletion infected with *T. gondii* resulted in a worse pregnancy than the control (Lao et al., 2015). Additionally, adoptive transfer of Treg cells to mice could improve adverse pregnancies of the animals (Liu et al., 2014). All of these investigations strongly suggest that the imbalance of immune tolerance at the maternal–fetal interface, in addition to direct parasite invasion, might be involved in the occurrence of abnormal pregnant consequences. To characterize the role of ROP16_I_ molecule in the pathogenicity of virulent type I strain, we generated *T. gondii* RHΔ*rop16* utilizing CRISPR/Cas9 technology to decipher the possible influence of ROP16_I_-deficient parasite infection on and the pathophysiology in the abnormal pregnancies. Our results uncovered that infection of mice with RHΔ*rop16* strain cause exacerbated adverse pregnant outcomes in which type I dominant response and enhanced pro-inflammatory cytokine expression were present in placenta tissues. The data provide further evidence that ROP16_I_ plays a potentially protective role in maintaining physiological immune tolerance during pregnancy, and other parasite-derived effectors might be associated with the M1–Th1 biased response at the maternal–fetal interface and the adverse pregnant outcomes.

## Materials and Methods

### Parasite Collection

*Toxoplasma gondii* RH WT and RHΔ*rop16* tachyzoites were cultured in human foreskin fibroblast (HFF) cells.

### *T. gondii* Infection in Pregnant Mice

The 8- to 10-week-old male and 6- to 8-week-old female C57BL/6 mice were raised for 1 week after purchase to adapt to the animal center environment with normal feeding and drinking at room temperature. The female and male mice were caged overnight at a 2:1 ratio. The female mice were examined for the vaginal plug as gestation day 0 (GD0). All pregnant mice were randomly divided into three groups: control group, RH WT group, and RHΔ*rop16* group, with seven mice in each. All of the experimental procedures were performed in accordance with the Institutional Review Board of AMU Institute of Biomedicine AMU (permit No: AMU26-081108). The studies were performed in licensed Biosafety II Laboratory. The RH WT and RHΔ*rop16* viable tachyzoites were harvested from HFF cells and counted with an Improved Neubauer counting board and diluted to 2 × 10^3^/ml. On gestation day 8 (GD8), mice of RH WT and RHΔ*rop16* groups were intraperitoneally injected with 200 μl of PBS containing 400 wild type and Δ*rop16* tachyzoites, respectively. The control animals were only given equal volume of PBS solution. On GD14, the animals were euthanized, blood was taken, and placentae, fetuses, uterus, and spleens were aseptically removed. The fetuses and placentae were photographed and weighed, and the number of resorbed fetuses was calculated. The resorptivity was determined by the small size, hemorrhagic appearance, and necrotic placenta and embryos. The ratio of the resorption to the total fetuses was recorded as a percentage of fetal loss. The sera were obtained by centrifugation (4,000 rpm, 10 min, 4°C) for ELISA detection.

### Pathology of Placental Tissues

Mice were randomly selected into three groups, with seven in each, for placental fixation with 4% paraformaldehyde, paraffin-embedded sections and hematoxylin–eosin staining. The hyperemia of placentae was observed by histopathological analyses. All mice in each group were photographed, with five photos for sections of each animal.

### Collection of Macrophages From Uterus and Placental Tissues

The uterus and placental tissues were quickly excised in a culture dish after removal, followed by digestion with 0.5% BSA, 5 mg/ml Collagenase IV, and HEPES (10 mM) (St. Louis, MO, United States) in RPMI 1640 solution (Montreal, QC, Canada), and slightly shaken at 37°C for 30 min at 150 rpm. Subsequently, the suspended cells were filtered through sterile nylon mesh. Macrophages were obtained by density gradient centrifugation at 25/50% Percoll (GE Healthcare Life Sciences).

### Isolation of Control Placental Macrophages and Infection of *T. gondii in vitro*

Macrophages in uterus and placental tissues from control pregnant mice were harvested according to the methods previously described and 2 × 10^6^ cells/well were cultured in 12-well plates. Macrophages were infected with 2 × 10^6^ RH WT and RHΔ*rop16* parasites, and the control group remained uninfected. The cells were cultured in an incubator at 37°C for 24 h to obtain macrophages. The macrophages were subjected to detection of the mRNA and protein expression of iNOS and Arg-1 and examination of phosphorylation of p38α mitogen-activated protein kinase (p38α MAPK).

### Western Blotting

Placental tissues of all mice and macrophages infected with the parasite were lysed using protein lysate (RIPA: PMSF = 100: 1), followed by centrifugation at 12,000 × *g* for 15 min at 4°C. The denatured proteins were separated by 10% separation gel in SDS-PAGE and transferred to NC membrane followed by blocking in 5% of milk on a shaker for 90 min. The NC membrane was incubated with primary antibodies against β-actin, Arg-1, and iNOS for placental tissues and additional incubation of p-p38α MAPK (Abcam, United Kingdom) for the macrophages infected with the parasite (both had 1:5000 dilution) at 4°C overnight. The anti-β-actin was used as the control. The NC membrane was washed with TBST three times, followed by the horseradish peroxidase-labeled secondary antibody incubation with slight shaking for 90 min. The specific bands were visualized by enhanced chemiluminescence. All experimental data were analyzed by ImageJ 1.46 software.

### Measurement of Macrophage Polarization by Flow Cytometry

Macrophages were obtained from the uterus and placental tissues of all mice and 2 × 10^6^ cells with 5 μl of mouse sera were added to each tube and let stand at 4°C for 30 min. The cells were labeled with anti-F4/80-BV421, anti-CD80-PE/cy5, anti-CD86-PE, and anti-CD206-AF647 (New York, BD, United States) at 4°C for 30 min in the dark. The cell surface markers were fixed after washing twice by PBS. All labeled samples were quickly detected by flow cytometry (FCM).

### Isolation and Culture of Splenocytes

The spleen tissues were taken out from the pregnant mice of three groups, and half of the tissues were mashed on a sterile nylon mesh to obtain spleen cells. Simultaneously, 2–3 ml of 1 × ACK Lysis buffer was added to the spleen cells in a tube for disrupting erythrocytes. Finally, the lysis was stopped with PBS, and the erythrocyte debris was removed by centrifugation at 2,500 rpm for 5 min, and the spleen cells were isolated and cultured at 37°C for 5 h under stimulation with ionomycin (1 mg/ml) and PMA (20 ng/ml) (St. Louis, MO, United States), and then centrifuged (4,000 rpm × 10 min at 4°C) to obtain supernatants for ELISA detection. The remaining spleen tissues were stored at −80°C for RNA extraction.

### Real-Time PCR for Detection of Cytokines

Total RNAs were extracted from spleens, placentae, and macrophages infected with the parasite and then reversed into cDNAs according to the Takara Kit instructions (Takara, Japan). Quantitative detection of IL-12, IL-10, TNF-α, TGF-β1, iNOS, and Arg-1 of placentae was performed with SYBR-Green Premix Ex Taq kit (Takara, Japan). Spleen tissues were only detected for IL-12, IL-10, TNF-α, and TGF-β1. The parasite-infected macrophages were detected for iNOS and Arg-1. The primers used in the qRT-PCR are listed in Table 1. The measurements were completed with Roche Applied Science Light Cycler TM 480 instrument. The results were normalized by β-actin and the results were calculated using the 2^–ΔΔCt^ method.

**TABLE 1:** The primers used for qRT-PCR.

**Primers**	**Forward primer (5′–3′)**	**Reverse primer (5′–3′)**
TNF-α	ACGGCATGGATCTCAAAGAC	GTGGGTGAGGAGCACGTAGT
iNOS	CACCTTGGAGTTCACCCAGT	ACCACTCGTACTTGGGATGC
Arg-1	CTCCAAGCCAAAGTCCTTAGAG	AGGAGCTATCATTAGGGACATC
IL-12	GATGTCACCTGCCCAACTG	TGGTTTGATGATGTCCCTGA
IL-10	GCTCCTAGAGCTGCGGACT	TGTTGTCCAGCTGGTCCTTT
TGF-β1	CTGGATACCAACTACTGCTTCAG	TTGGTTGTAGAGGGCAAGGACCT
β-actin	CTGTCCCTGTATGCCTCTG	ATGTCACGCACGATTTCC

*IL, interleukin; iNOS, inducible nitric synthase; Arg-1, arginase-1; TNF, tumor necrosis factor; TGF-β1, transforming growth factor beta 1.*

### ELISA for Cytokines

The placental tissues were weighed and ultrasonicated in the ratio of 1 mg/10 μl PBS (at 3-s intervals) and centrifuged at 12,000 × *g* at 4°C for 10 min. The supernatants were extracted and cytokines of TNF-α (Cusabio, Houston, TX, United States) and IL-12 (Elabscience, Wuhan, China) in the supernatants of placental homogenates, spleen cell culture, and sera were detected by ELISA. The examinations of all specimens were performed three times and the OD values were measured at a wavelength of 450 nm. The concentration of TNF-α and IL-12 was calculated by plotting a standard curve according to the manufacturer’s instruction.

### Statistical Analysis

The data were presented as the mean ± SD and were statistically analyzed by one-way ANOVA after precheck for normal distribution and homogeneity of variances, and *p* < 0.05 or *p* < 0.01 indicated statistical significance. Analyses of experimental data and graphic production were obtained by GraphPad Prism Software.

## Results

### RHΔ*rop16* Strain Infection Caused Severe Abnormal Pregnancy

**FIGURE 1:**
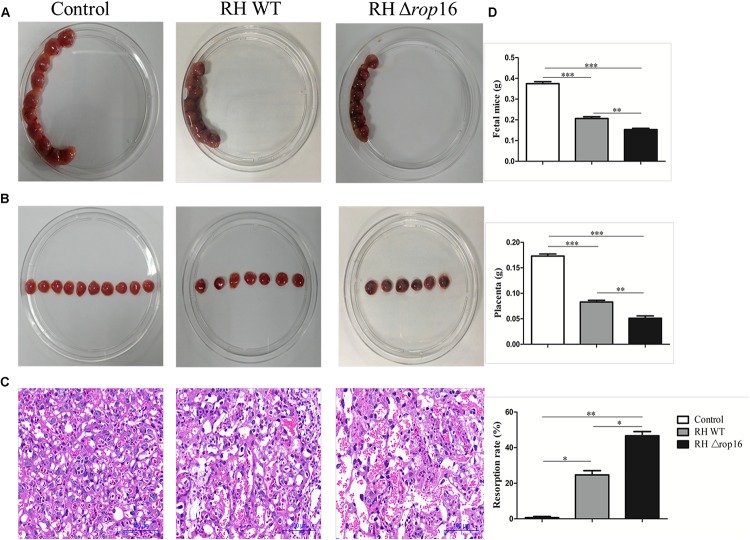
The occurrence of adverse pregnancy outcomes caused by *Toxoplasma gondii* infection. Fetuses and placentae of the control mice developed normally. The less number of fetuses and the more serious hemorrhage of placentae were seen in the mice of the RHΔ*rop16*-infected group when compared with the RH WT group **(A,B)**. H&E staining showed deteriorated hyperemia of placentae caused by RHΔ*rop16* strain infection **(C)**. The weights of fetuses and placentae and fetus resorption of each group were measured after 6 days of infection. The ratio of resorption to total fetuses was calculated as the percentage of fetal loss **(D)**. Each group contained seven mice. The results were presented as mean ± SD and were analyzed by one-way ANOVA (^∗^*p* < 0.05, ^∗∗^*p* < 0.01, ^∗∗∗^*p* < 0.001).

Pregnancy outcomes were examined at GD14 (i.e., 6 days post-infection). The pregnant mice infected with RHΔ*rop16* strain showed a clinical manifestation of wilting and arching, accompanied with aggravated fetal resorption and placental hemorrhage when compared with the RH WT group (Figures 1A,B). The H&E staining of placenta tissues exhibited more severe hyperemia in the RHΔ*rop16*-infected group than that in the RH WT group (Figure 1C). The weights of placentae and fetuses in mice of the RHΔ*rop16-*infected group were notably reduced compared with the RH WT group. Accordingly, the resorptivity of the fetuses in the RHΔ*rop16*-infected group significantly increased (Figure 1D).

### Expression of iNOS, Arg-1, and p-p38α MAPK Varied in Placental Macrophages Infected With RHΔ*rop16* Strain *in vitro*

**FIGURE 2:**
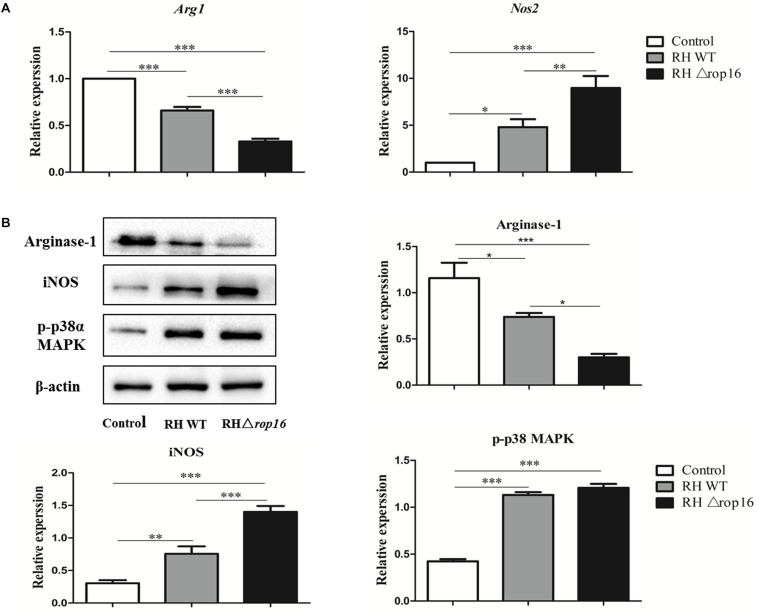
*In vitro* expression of Arg-1, iNOS, and p-p38α MAPK of placental macrophages from control pregnant mice following *T. gondii* infection. Placental macrophages of control pregnant mice were collected and infected with RH WT and RHΔ*rop16 in vitro* for 24 h. The mRNA and protein expressions of Arg-1 and iNOS of macrophages were examined by qRT-PCR **(A)** and Western blotting **(B)**. Phosphorylation of p38α MAPK was detected by Western blotting **(B)**. Each group contained seven mice. The results were given as mean ± SD and were analyzed by one-way ANOVA (^∗^*p* < 0.05, ^∗∗^*p* < 0.01, ^∗∗∗^*p* < 0.001).

To examine the direct impact of *T. gondii* ROP16_I_ on the bias of macrophages, we isolated primary macrophages from placental and uterine tissues of control pregnant mice. The cells were infected with RH WT and RHΔ*rop16* parasites, and cultured for 24 hr. The data showed that the expression of iNOS was significantly elevated while that of Arg-1 was diminished in the macrophages with RHΔ*rop16* infection when tested by qRT-PCR (Figure 2A) and Western blotting (Figure 2B). No significant difference of p-p38α MAPK expression, however, was noted between RH WT and RHΔ*rop16*-infected macrophages by Western blotting analysis (Figure 2B and Supplementary Figure S1).

### RHΔ*rop16* Strain Infection Modulated Synthesis of iNOS and Arg-1 in Placental Tissues *in vivo*

**FIGURE 3:**
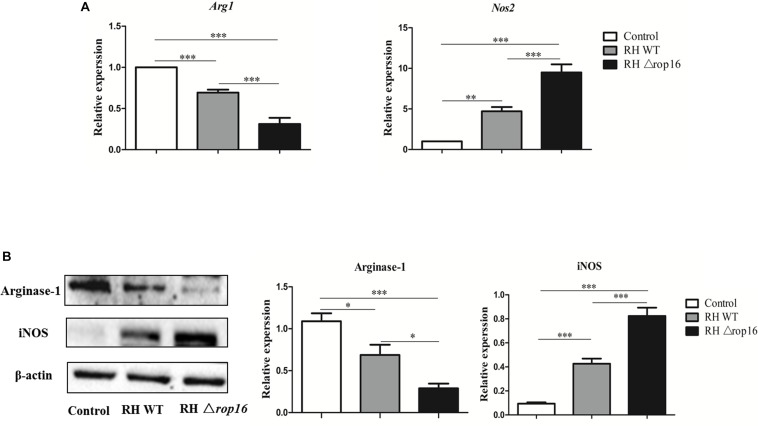
*In vivo* expression of Arg-1 and iNOS in placental tissues. Placental tissues were removed from three experimental groups. The mRNA and protein expressions of Arg-1 and iNOS of placental tissues were examined. The relative mRNA expressions of Arg-1 and iNOS were detected in placental tissues of mice infected with RHΔ*rop16* strain **(A)**. The corresponding results of protein expressions were also noted with Western blotting **(B)**. The results were presented as mean ± SD obtained from seven mice in each group and were analyzed by one-way ANOVA (^∗^*p* < 0.05, ^∗∗^*p* < 0.01, ^∗∗∗^*p* < 0.001).

Expression of iNOS and Arg-1 in placental tissues in mice of three groups was assayed by qRT-PCR (Figure 3A) and Western blotting (Figure 3B). As shown in Figure 3, the upregulated expression of iNOS mRNAs and downregulated expression of Arg-1 mRNAs were observed in the RHΔ*rop16*-infected group, when compared to the RH WT group. Correspondingly, the protein level of iNOS was remarkably elevated but Arg-1 was reduced when detected by Western blotting in RHΔ*rop16*-inoculated mice.

### The Phenotypes of Mouse Placental Macrophages Altered Following RH Δ*rop16* Strain Infection

**FIGURE 4:**
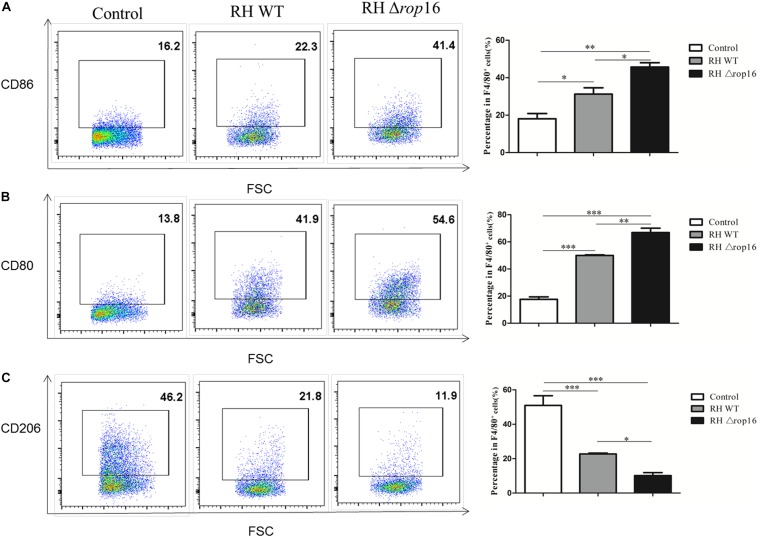
Expression of CD86, CD80, and CD206 markers at the maternal–fetal interface. Placental macrophages were removed from three experimental groups. A high expression of CD206 but low expression of CD80 and CD86 of macrophages were noted in control pregnant mice by flow cytometry, while RHΔ*rop16* parasite infection stimulated high-level expressions of CD86 **(A)** and CD80 **(B),** and low level of CD206 **(C)** in the placental macrophages of pregnant mice. Experimental and control group each contain seven mice. The results were given as mean ± SD and were analyzed by one-way ANOVA (^∗^*p* < 0.05, ^∗∗^*p* < 0.01, ^∗∗∗^*p* < 0.001).

Density gradient centrifugation was used to isolate placental macrophages for detection of polarization of the macrophages in placental tissues of mice infected with RHΔ*rop16* strain. The surface markers of CD86 and CD80 on M1 macrophages and CD206 on M2 cells were detected by FCM. The results showed that RHΔ*rop16* infection induced higher expression of CD86 (Figure 4A) and CD80 (Figure 4B) but lower expression of CD206 (Figure 4C) on the surface of placental macrophages, when compared to the RH WT infection group.

### RHΔ*rop16* Infection Evoked Th1-Biased Response in Spleens, Placentae, and Sera

**FIGURE 5:**
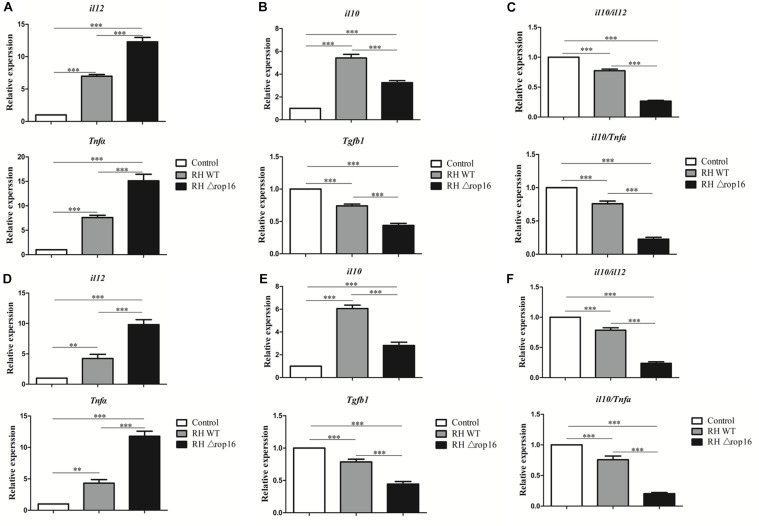
Expression of IL-12, TNF-α, IL-10, and TGF-β1 in spleens and placentae of mice with *T. gondii* infection. Spleen tissues and placentae were taken from three groups of experimental mice. The mRNA transcriptions of pro-inflammatory cytokines of IL-12 and TNF-α and anti-inflammatory cytokine of IL-10 and TGF-β1 were examined in spleen tissues **(A,B)** and placentae **(D,E)**. The ratio of mRNA transcriptions of IL-10 to IL-12 and IL-10 to TNF-α was calculated in spleen tissues **(C)** and placentae **(F)**. Each sample was performed in triplicate. The results were presented as mean ± SD from seven mice of each group (^∗∗^*p* < 0.01, ^∗∗∗^*p* < 0.001).

**FIGURE 6:**
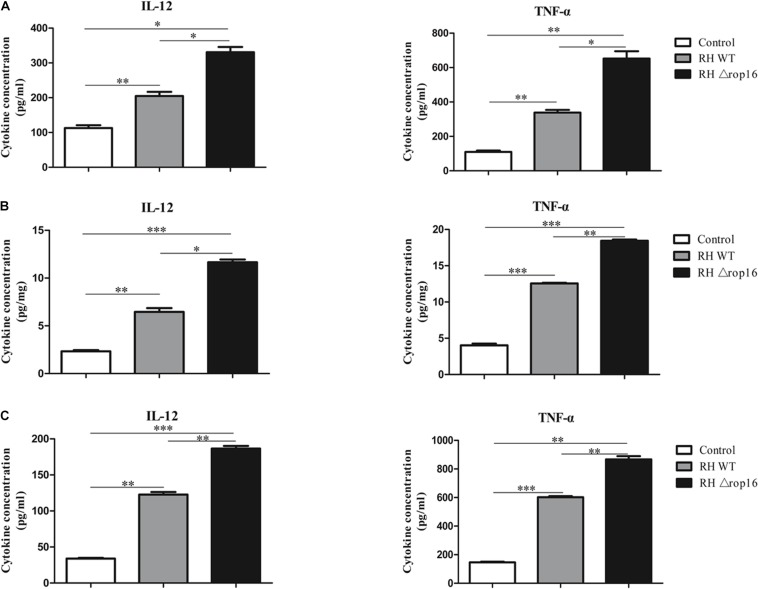
IL-12 and TNF-α expression in splenocytes, placental tissues, and sera. The supernatants of splenocytes and placental homogenates were taken from three experimental groups. The sera were obtained by centrifugation (4,000 rpm, 10 min, 4°C) from the three groups. Expressions of IL-12 and TNF-α in the supernatants of splenocytes (pg/ml) **(A)**, placental homogenates (pg/mg) **(B)**, and sera (pg/ml) of pregnant mice **(C)** were evaluated with ELISA. Each sample was performed in triplicate. Each group contained seven animals. The results were presented as mean ± SD (^∗^*p* < 0.05, ^∗∗^*p* < 0.01, ^∗∗∗^*p* < 0.001).

Tissues of placentae and spleens were collected from three groups, and the mRNA expression of IL-12, TNF-α, IL-10, and TGF-β1 was measured by qRT-PCR. The results showed that the mRNA expression of IL-12 and TNF-α in the spleens (Figure 5A) and placentae (Figure 5D) were highly transcribed, but expression of TGF-β1 mRNA was dramatically dampened (Figures 5B,E) in the animals of RHΔ*rop16*-inoculated mice, when compared to the RH WT infection group. Unexpectedly, the level of IL-10 expression in the spleens (Figure 5B) and placentae (Figure 5E) was significantly elevated in either of the two infected groups compared to the control. However, the ratio of IL-10/IL-12 as well as IL-10/TNF-α in the spleens (Figure 5C) and placentae (Figure 5F) of the RHΔ*rop16*-infected mice remained low in comparison to the control mice. Additionally, the ELISA assay revealed the significantly increased levels of pro-inflammatory cytokines of IL-12 and TNF-α in the supernatants of splenocytes and placental homogenates, and sera as well, in RHΔ*rop16*-infected mice (Figures 6A–C).

## Discussion

It has been known that numerous immune cells contribute to maintaining the equilibrium state of the maternal–fetal immune interface. Macrophages are the first line of defense in innate immunity. During normal pregnancy, macrophages also perform functions such as angiogenesis, tissue repair, and immune tolerance (Mor and Abrahams, 2003). Alternatively activated macrophages (M2 macrophages), uterine/decidual NK cells, as well as Tregs are crucial cell populations that contribute to the immune tolerance at the maternal–fetal interface and keep the fundamental physiological processes of pregnancy by secreting large amounts of cytokines and chemokines (Lidstrom et al., 2003; Kalkunte et al., 2008; Co et al., 2013; Gaynor and Colucci, 2017). A growing body of evidence indicated that pregnancy failure induced by *T. gondii* infection is associated with the altered maternal immunity (Zhang et al., 2012; Zhao et al., 2017). It has been reported that the LILRB4 expression of decidual macrophages decreased after infection with *T. gondii*, which induced polarization of classically activated macrophages (M1 macrophages) and inhibited the immune bias of M2 direction, leading to adverse pregnancy outcomes (Li et al., 2017). In addition, trophoblast cells are the only cells that are in direct contact with the maternal immune system and play an important part in embryo implantation and maternal–fetal immune tolerance. Apoptosis of trophoblast cells induced by inflammation may directly impact the microenvironment of maternal–fetal interface, resulting in abnormal pregnant outcomes such as miscarriage (Reister et al., 2001). Additional investigations showed that *T. gondii* infection induced the activation of macrophages and their secretion of a variety of pro-inflammatory factors through oxidative stress-mediated apoptosis of placental trophoblasts, more likely leading to adverse pregnancy events (Aschkenazi et al., 2002; Neale et al., 2003; Liu et al., 2013; Xu et al., 2015). Overall, any peripheral or local infection in the maternal side during the first trimester is prone to cause serious adverse pregnancy outcomes.

*Toxoplasma gondii* effectors of ROP16_I_ and GRA15_II_ are responsible for inducing macrophage polarizations and play a pivotal role in host innate immunity and directly affect the consequences of infection or disease. During invasion into host cells, *T. gondii* injects the rhoptry protein ROP16 into the host cytoplasm, which subsequently localizes to the parasitophorous vacuole membrane (PVM). We previously found that deletion of ROP16_I_ of either RH strain of type I or WH3 strain of type Chinese 1 did not affect the virulence of the parasite to mice (Wang et al., 2018), although ROP16 is believed as one of polymorphic and strain-dependent virulence factors (Saeij et al., 2006). Studies have shown that ROP16_I_, rather than ROP16_II_, serves as a central regulator of parasite replication and transmission at early phase of infection and drives the macrophages to M2 polarization. Contrarily, GRA15_II_, one of the key molecules secreted by dense granules of type II strains, evokes strong M1 macrophage polarization by directly activating NF-κB and facilitating a high expression of IL-12 and iNOS that are involved in the subsequent Th1 predominant immune response and the pro-inflammatory reaction (Jensen et al., 2011; Rosowski et al., 2011). We previously demonstrated that *T. gondii* strains of type Chinese 1, distinct from the strains of archetypal I, II, and III strains prevalent in the other continents of the world, carry both ROP16_I_ and GRA15_II_ alleles. Deletion of ROP16_I_ of the Chinese 1 WH3Δ*rop16*, with GRA15_II_ genetic background, resulted in remarkably adverse pregnancy outcomes in mice (Wang et al., 2018). However, the pathogenic feature of the RHΔ*rop16* strain of type I, with neither ROP16_I_ nor GRA15_II_ background, in *T. gondii*-induced aberrant pregnancy remains unknown. Thus, we examined the pathology of adverse pregnant outcomes caused by ROP16_I_-deficient *T. gondii* (RHΔ*rop16*) and the mechanisms by which the parasite infection subverts the immune tolerance at the maternal–fetal interface in the murine model. We noted that all of the mice infected with *T. gondii* had clinical manifestations of wilting and arching; however, the RHΔ*rop16* strain caused the exacerbated abnormal pregnancy outcomes when compared with the RH WT strain, as evidenced by the lower number and reduced weights of placentae and fetuses, hemorrhage of placentae, and a high rate of fetal resorption. A previous study showed that deficiency of ROP16_I_ of the RH strain did not affect proliferation of the tachyzoites (Butcher et al., 2011), but no parasites were seen in the damaged placenta tissues of RHΔ*rop16*-infected mice (data not shown), suggesting that the pathology of placentae might be attributable to the biased M1–Th1 immune response following infection. Additionally, our data indicated that IL-12 and TNF-α were significantly increased whereas TGF-β1 was decreased in the RHΔ*rop16*-infected animals; the ratios of IL-10/IL-12 and IL-10/TNF-α of the RHΔ*rop16*-infected mice declined significantly compared to those of the RH WT group, indicative of the Th1-polarized response in RHΔ*rop16* infection. Moreover, placental macrophages tended to be driven to M1 skewing after 6 days of *T. gondii* infection. The results suggest that the pro-inflammatory factors in the placentae induced by RHΔ*rop16* infection might be involved in the aberrant pregnancy of mice, which is attributable to the deletion of ROP16_I_ of the RH strain.

Theoretically, any negative impact of infections on immune tolerance at the maternal–fetal microenvironment may lead to abnormal pregnancy. It has been demonstrated that *T. gondii* type II strains are frequently found in human infection of Europeans (Wujcicka et al., 2014) and are responsible for pregnant failure (Liu et al., 2018), which might be related to GRA15_II_-induced M1–Th1 dominant response. Genetic structures of *T. gondii* in South America are complex, and these non-archetypal strains may cause serious consequences of pregnancies (Rajendran et al., 2012). It is speculated that multiple parasite-derived molecules, in addition to the direct invasion of parasites, may contribute to the adverse pregnant consequences. We here noted that mice infected with the RH strain with *rop16* deletion caused deteriorated pregnant outcomes, suggesting a protective influence of ROP16_I_ on normal pregnancy. Similar reports were given by Jensen et al. (2011) that ROP16_I_ might have an ameliorative effect on *T. gondii*-induced ileitis. Additionally, our studies strongly suggested that other *T. gondii* effectors, besides GRA15_II_, might be involved in the pathology process of pregnancy. This speculation is supported by the previous investigations that demonstrated that GRA24, a member of GRAs family with 542 amino acids similar to GRA15_II_ of *T. gondii* in function, can bypass the classical MKK3/MKK6 and MKK4 pathways to directly bind to the p38α MAPK, leading to activation and nuclear translocation of the host kinase and increasing IL-12 secretion and M1 macrophages polarization (Kim et al., 2005; Braun et al., 2013). Further approaches are needed to identify the effectors of *T. gondii* that induce IL-12 production and M1 polarization in the occurrence of abnormal pregnancy.

Taken together, the RH strain of type I *T. gondii* with ROP16_I_ deletion may cause severe abnormal pregnant outcomes in mice, with the feature of M1 macrophage phenotype and Th1 dominant response in system and placental tissues, suggesting that ROP16_I_ might be a protective factor and other parasite-derived molecules may be involved in the pathology process of pregnancy failure.

## Data Availability Statement

All datasets generated for this study are included in the article/Supplementary Material.

## Ethics Statement

Ethical permission was obtained from the Institutional Review Board of AMU Institute of Biomedicine AMU (Permit No. AMU26–081108).

## Author Contributions

JS, WW, and WC elaborated and designed the study, and drafted the manuscript. WC, CW, and TX performed the experiments. QL analyzed the data. All authors have read and approved the final version of the manuscript.

## Conflict of Interest

The authors declare that the research was conducted in the absence of any commercial or financial relationships that could be construed as a potential conflict of interest.

## Supplementary Material

FIGURE S1 The expression of β-actin (40KD), Arg-1(36KD), and p-p38 MAPK (40KD) of placental macrophages infected with the parasite were examined by Western blotting.
