# Supplementary figures and images for: Toxoplasma gondii ROP16I Deletion: The Exacerbated Impact on Adverse Pregnant Outcomes in Mice
Source: Front Microbiol. 2020 Jan 31;10:3151. doi: 10.3389/fmicb.2019.03151 (PMC7005636; doi:10.3389/fmicb.2019.03151)

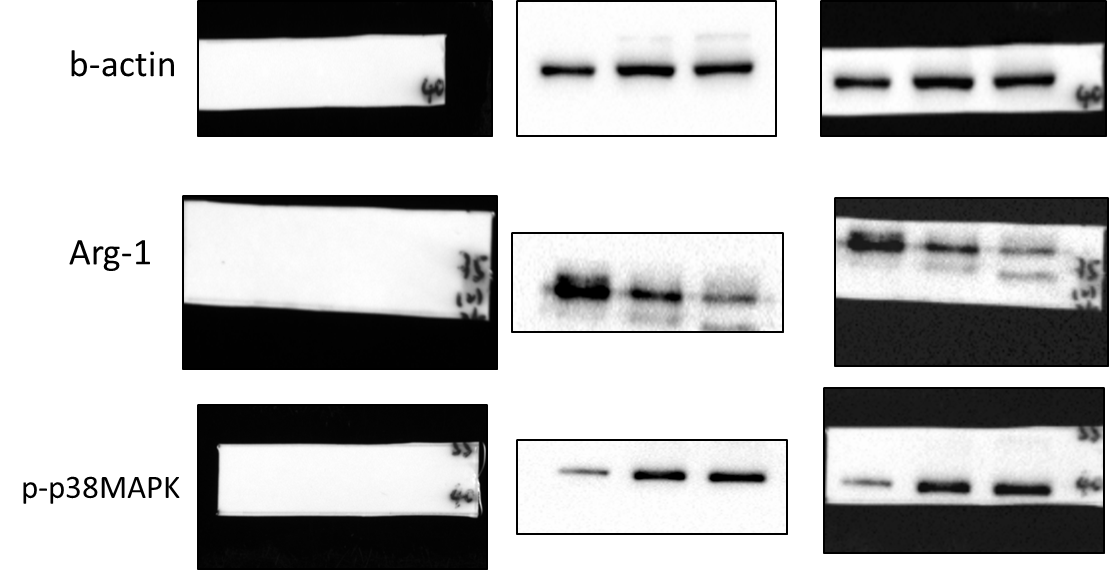

Supplement: FIGURE S1 — The expression of β-actin (40KD), Arg-1(36KD), and p-p38 MAPK (40KD) of placental macrophages infected with the parasite were examined by Western blotting. [file Image_1.TIF]
